# Gender Differences in Low-Molecular-Mass-Induced Acute Lung Inflammation in Mice

**DOI:** 10.3390/ijms22010419

**Published:** 2021-01-03

**Authors:** Yifang Xie, Dehui Xie, Bin Li, Hang Zhao

**Affiliations:** Collaborative Innovation Center of Yangtze River Delta Region Green Pharmaceuticals, Zhejiang University of Technology, Hangzhou 310014, China; 2111823025@zjut.edu.cn (Y.X.); 2111923051@zjut.edu.cn (D.X.); 2111923049@zjut.edu.cn (B.L.)

**Keywords:** low-molecular-mass hyaluronan (200 kDa), acute lung inflammation, gender differences, 17β-estradiol

## Abstract

Gender differences in pulmonary inflammation have been well documented. Although low molecular mass hyaluronan (LMMHA) is known to trigger pulmonary lung inflammation, sex differences in susceptibility to LMMHA are still unknown. In this study, we test the hypothesis that mice may display sex-specific differences after LMMHA administration. After LMMHA administration, male mice have higher neutrophil, cytokine, and chemokine counts compared to that of their female counterparts. Additionally, Ovariectomized (OVX) mice show greater LMMHA-induced inflammation compared to that of mice with intact ovaries. Injections of OVX mice with 17β-estradiol can decrease inflammatory responses in the OVX mice. These results show that ovarian hormones regulate LMMHA induced lung inflammation.

## 1. Introduction

Hyaluronan, or hyaluronic acid (HA), is a major component of an extracellular matrix with a structure made up of polymeric disaccharides d-Glucuronic acid and N-acetyl-d-glucosamine A linked by glucuronic acid bonds [1]. Rapid degradation of hyaluronic acid produces low-molecular-mass HA (LMMHA), which has pro-inflammatory effects and acts as an intracellular signal molecule in inflammation [2]. Patients with acute respiratory distress syndrome, idiopathic pulmonary fibrosis, chronic obstructive pulmonary disease, and asthma have significantly increased LMMHA [3,4,5,6]. 

LMMHA upregulates adhesion molecules in endothelial cells and induces production of pro-inflammatory cytokines in airway epithelial cells [7]. In vitro studies have shown that in response to LMMHA stimulation, macrophages are the main effector cells in producing pro-inflammatory chemokines and cytokines [7]. Neutrophils also play key roles in inflammatory pulmonary diseases [8,9]. Previously, we reported that intratracheal administration of LMMHA (200 kDa) causes neutrophil infiltration in mice lungs, which is associated with an increase in interleukin-6 (IL-6), chemokine (C-X-C motif) ligand 1 (CXCL-1), tumor necrosis factor-α (TNF-α), and chemokine (C-X-C motif) ligand 2 (CXCL-2) levels [10,11]. Our previous study suggests that phosphoinositide 3-kinase (PI3K)/Akt1 signaling plays a key role in LMMHA (200 kDa)-induced lung inflammation [12].

Both adults and children have sex-specific differences in organ injury. Morbidity and incidence of acute lung injury are higher in males [13,14,15,16]. Although the gender difference in pulmonary response is obvious in different animal models of experimental lung injury [17], it is unknown whether there is a difference in inflammatory responses between males and females after intratracheal administration of hyaluronan (200 kDa).

## 2. Results

### 2.1. Plasma Estradiol Concentration

Plasma estradiol concentration was measured in female mice (proestrus, estrus, metaestrus, and diestrus) and in Ovariectomized (OVX) mice and 17β-estradiol (E2) treated OVX mice. Plasma estradiol is at its maximum in the proestrus stage and reaches levels of ~60 pg/mL, but is reduced to ~20 pg/mL during estrus and diestrus. Estradiol was below the detection limit in OVX mice. Plasma estradiol levels increased to ~120 pg/mL in OVX mice that received E2 injections for 7 consecutive days.

### 2.2. Gender Differences after LMMHA Induces Lung Inflammation after Intratracheal Administration

**Figure 1:**
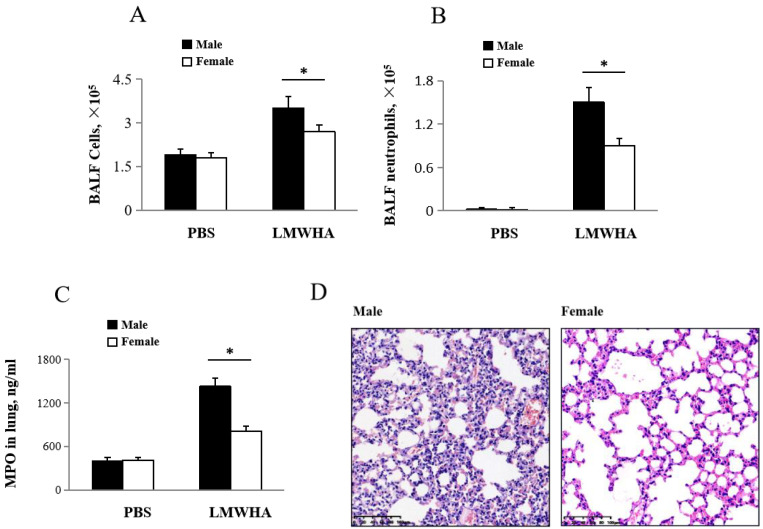
Male mice show greater inflammatory cell infiltration compared to females. 12 h after intratracheal administration of low molecular mass hyaluronan (LMMHA) (200 kDa, 65 mg/kg), (**A**) total cell and (**B**) neutrophil counts were performed on BALF. After BALF was performed, (**C**) MPO of whole lung homogenates was measured, (**D**) Lung sections were stained with H&E, original magnification 200×. Values are means ± SEM from at least five individual animals. Significant differences are indicated by *, *p* < 0.05.

Hyaluronic acid fragments (of a molecular mass of 200 kDa) are common at inflammatory sites in the lung and can induce lung inflammation. LMMHA (200 kDa) activates and attracts neutrophils, which lead to lung inflammation. An intratracheal dose of LMMHA (200 kDa, 65 mg/kg) increases total cell (Male: 3.5 × 10^5^ ± 0.4 × 10^5^/mL, Female: 2.7 × 10^5^ ± 0.22 × 10^5^/mL) and neutrophil counts (Male: 1.5 × 10^5^ ± 0.11 × 10^5^/mL, Female: 0.9 × 10^5^ ± 0.08 × 10^5^/mL) in BALF in both males and females (Figure 1A, B). To further quantify neutrophil sequestration, MPO activity, which reflects the parenchymal infiltrated of neutrophils and macrophages in the lung tissue [18], was measured. These results show an increase in MPO activity in LMMHA-treated mice 12 h after LMMHA administration (Figure 1C) in both male and female mice, with a greater increase in male mice (Male: 1429 ± 116 ng/mL, Female: 810 ± 70 ng/mL). Compared to male mice, female mice show reduced thickening of the alveolar septum and reduced infiltration of neutrophils (Figure 1D). 

### 2.3. Differentially Expressed Cytokine Levels in Mice Lungs Induced by LWMHA

**Figure 2:**
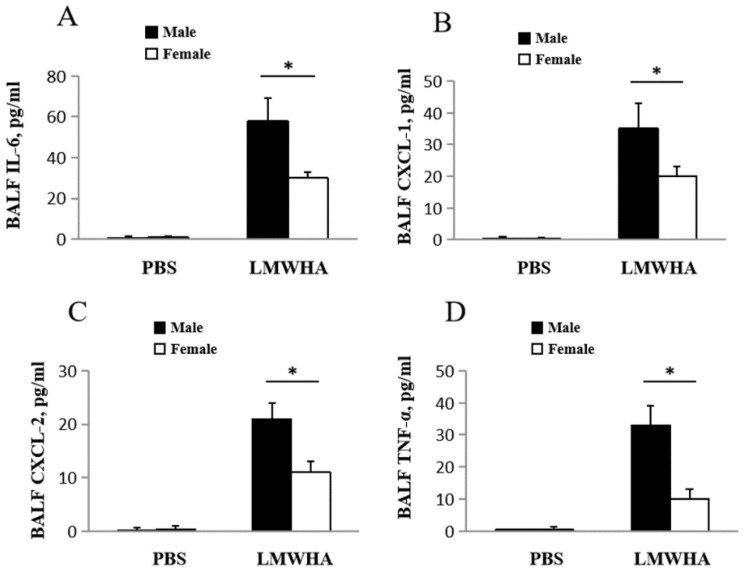
Male mice have a higher level of inflammatory cytokine production compared to that of females 12 h after intratracheal administration of LMWHA (200 kDa, 65 mg/kg). (**A**) IL-6, (**B**) CXCL-1, (**C**) CXCL-2, and (**D**) TNF-α in BALF were analyzed. Values are means ± SEM from at least five individual animals. Significant differences are indicated by *, *p* < 0.05.

Pro-inflammatory chemokine and cytokine levels in BALF were determined to further quantify LMMHA-triggered inflammatory response. IL-6, an inflammatory marker, delays spontaneous apoptosis of neutrophils and enhances the aggregation and adhesion of neutrophils [9]. The levels of IL-6 (Male: 58 ± 11 pg/mL, Female: 30 ± 3 pg/mL) (Figure 2A), CXCL-1 (Male: 35 ± 12 pg/mL, Female: 20 ± 3 pg/mL) (Figure 2B), and TNF–α (Male: 33 ± 6 pg/mL, Female: 10 ± 4 pg/mL) (Figure 2C) were higher in male mice after exposure to LMMHA compared to that of female mice. CXCL-2 is a member of the CXC chemokine family and plays a critical role in the recruitment of neutrophils to tissue damage, infection, and wound recovery sites [19,20,21]. CXCL-2, secreted by monocytes such as peritoneal and alveolar macrophages [22,23], belongs to the CXC chemokine subfamily (α-chemokines) which are chemoattractants. Previous studies have also shown that CXCL-2 helps recruit neutrophils in the development of inflammation and tissue injury [24,25]. Our results show (Figure 2D) that CXCL-2 activities are dramatically higher in the male group (Male: 21 ± 5 pg/mL, Female: 11 ± 2 pg/mL). Hence, intratracheal administration of LMMHA leads to clear gender differences in mice lung inflammation.

### 2.4. Estradiol Effects on LMMHA-Induced Lung Injury

**Figure 3:**
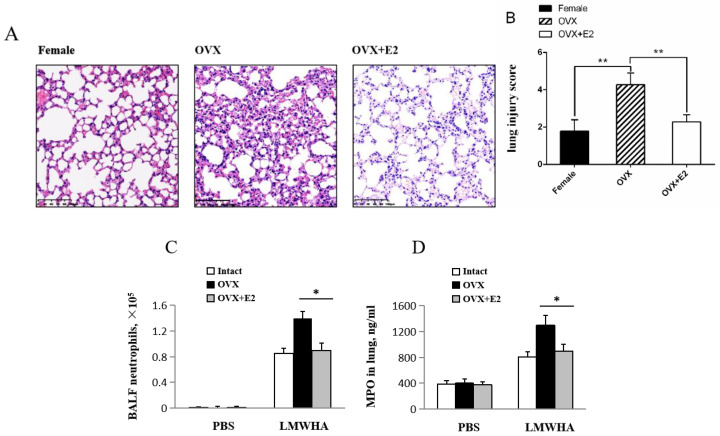
Effects of OVX mice treated with 17β-estradiol (E2) on inflammatory cell infiltration. After a recovery period of at least two weeks, each OVX mouse received daily subcutaneous injection of either vehicle (sesame oil, 2 mL/kg BW) or E2 (twice a day, 3 μg/kg BW) for one week. (**A**) Lung sections stained with H&E, original magnification 200×. Values are means ± SEM from at least five individual animals, (**B**) Lung injury score, (**C**) total cell counts, and (**D**) neutrophil counts were performed on BALF 12 h after intratracheal administration of LMWHA (200 kDa, 65 mg/kg). Significant differences are indicated by *, *p* < 0.05.

To determine the role of estrogen in lung inflammation, we evaluated if E2 can mimic regulatory functions of ovariectomized mice in acute lung injury. These results show that compared to OVX mice, alveolar septum thickening and neutrophil infiltration is reduced in those mice that have an E2 supplement (Figure 3A). Lung injury score was significantly decreased in E2 treated OVX mice on quantal scoring of histological lung injury severity (Figure 3B). OVX mice treated with E2 show significantly reduced neutrophil counts in BALF compared to that of LMMHA-treated OVX mice (Intact: 0.85 × 10^5^ ± 0.08 × 10^5^/mL, OVX: 1.4 × 10^5^ ± 0.1 × 10^5^/mL, OVX + E2: 0.9 × 10^5^ ± 0.11 × 10^5^/mL) 12 h after LMMHA administration (Figure 3C). OVX mice treated with E2 demonstrate less intense MPO activity in the lung tissue compared with OVX mice (Intact: 815 ± 75 ng/mL, OVX: 1300 ± 150 ng/mL, OVX + E2: 900 ± 100 ng/mL) (Figure 3D). 

**Figure 4:**
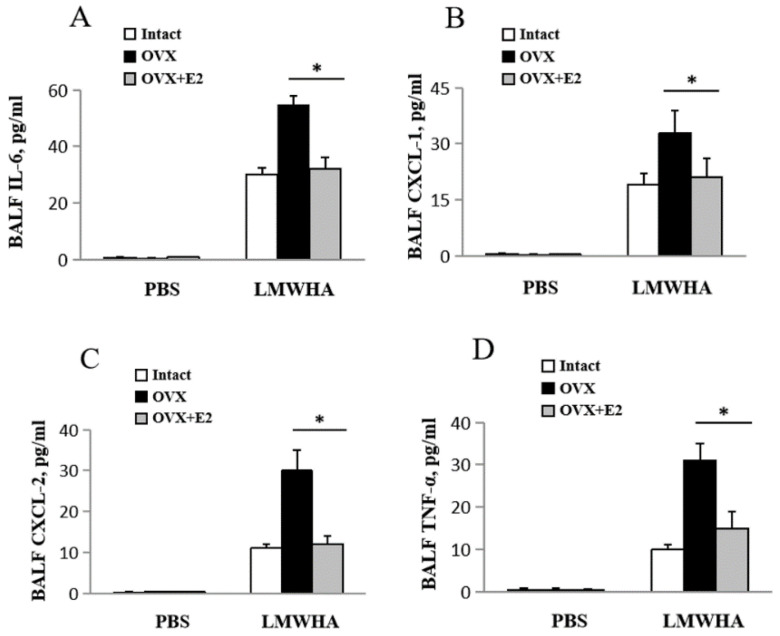
Effects of OVX mice treated with 17β-estradiol (E2) on inflammatory cytokine production. After a recovery period of at least two weeks, each OVX mouse received daily subcutaneous injection of either vehicle (sesame oil, 2 mL/kg BW) or E2 (twice a day, 3 μg/kg BW) for one week. (**A**) IL-6, (**B**) CXCL-1, (**C**) CXCL-2, and (**D**) TNF-α in BALF were analyzed 12 h after intratracheal administration of LMWHA (200 kDa, 65 mg/kg). Values are means ± SEM from at least five individual animals. Significant differences are indicated by *, *p* < 0.05.

Compared to OVX mice, E2-treated OVX mice show significantly reduced lung IL-6 levels (Intact: 30 ± 2.5 pg/mL, OVX: 55 ± 3 pg/mL, OVX + E2: 32 ± 4 pg/mL) (Figure 4A), CXCL-1 (Intact: 19 ± 3 pg/mL, OVX: 33 ± 6 pg/mL, OVX + E2: 21 ± 5 pg/mL) (Figure 4B), CXCL-2 (Intact: 11 ± 1 pg/mL, OVX: 30 ± 5 pg/mL, OVX + E2: 12 ± 2 pg/mL) (Figure 4C), TNF-α (Intact: 10 ± 1 pg/mL, OVX: 31 ± 4 pg/mL, OVX + E2: 15 ± 4 pg/mL) (Figure 4D) 12 h after LMMHA administration.

## 3. Discussion

In this study, we demonstrate that male mice develop greater lung inflammation than female mice after intratracheal administration of LMMHA. Ovariectomized females exhibit increased inflammation compared to females with intact ovaries. Furthermore, 17β-estradiol injection into ovariectomized female mice reduces inflammation. Therefore, 17β-estradiol may be a key contributor to disparate gender effects in LMMHA induced inflammation. These findings agree with previous clinical studies in which female mice have reduced morbidity and mortality due to ischemia/reperfusion, trauma, sepsis, and shock, which are common risk factors for ALI [13,14,26,27]. Administration of 17β-estradiol limits neutrophil infiltration and subsequent tissue destruction in liver trauma [28,29], carrageenan-induced pleurisy, tracheal LPS damage [17,30], and OVA-induced allergic reaction [31]. Since neutrophils contribute to the acute phase of inflammation and recruit other innate immune system players, 17β-estradiol suppression of neutrophil activity could have impressive effects on immune response. 

Neutrophils are key mediators of inflammatory response, particularly in LMMHA-induced lung injury [10]. The process of neutrophil extravasation involves multiple subsequent steps of margination and rolling, adhesion, diapedesis, and migration. The steps of margination and rolling are mediated by E-selection and P-selectin on the vasculature, which bind to Sialyl Lewis on the neutrophil. This interaction allows leukocytes to travel to areas of need in the body before tightly binding to endothelial cells (through intracellular adhesion molecule 1, ICAM-1) and traveling between endothelial cells to the site of injury. Previous studies have shown that P-selectin is at its lowest during the luteal phase in women and that an intramuscular 17β-estradiol injection significantly lowers soluble P-selectin levels in healthy males [32]. Ovariectomized mice demonstrate an increase in neutrophil accumulation compared to that of cycling animals, and 17β-estradiol inhibits LMMHA induced lung inflammatory responses. Our study implicates endogenous 17β-estradiol in the regulation of neutrophil infiltration by controlling the levels of P-selectin and ICAM-1 in lung tissue injury.

Neutrophils are removed by apoptosis and macrophage phagocytosis from areas of tissue injury, leading to resolution of inflammation [33]. This process is particularly important in the resolution of lung inflammation. Disruption to the neutrophil apoptosis process can lead to acute respiratory distress syndrome (ARDS) and sepsis [34]. Neuronal, cardiac, and renal ischemia are additionally subject to sex-specific differences in apoptosis [35,36,37]. 17β-estradiol may also inhibit the accumulation of LMMHA treated neutrophils in lung by impairing their survival.

Differences between LMMHA-induced neutrophil infiltration in males and females could be the result of sex specific alterations in the neutrophil estrogen receptor (ER) phenotype: expression of ER subtypes depends on exposure of neutrophils to estrogen. Men and premenopausal women express ERα and ERβ neutrophil subtypes. Ovulation in women increases estrogen and upregulates ERα and ERβ. The follicular phase of the menstrual cycle is associated with low estrogen, but in vitro incubation of follicular phase neutrophils with 17β-estradiol leads to similar upregulation of the ERα and ERβ subtypes [38]. Interestingly, only the ERα subtype is upregulated when neutrophils from men are incubated with 17β-estradiol, which suggests sex-differences of ERα and ERβ subtypes expression [38]. These differences may explain why neutrophils from men have reduced neutrophil adhesion [39]. ERα is an important facilitator of the inflammatory response in many types of tissue through its effects on estrogen such as in alveolar regeneration [40,41,42,43,44]. On the other hand, ERβ plays a role in trauma-hemorrhage induced lung injury [45], but ERβ agonists do not impact the lung inflammatory response [46]. Further studies are needed to determine how ERα and ERβ lead to these effects on LMMHA-treated neutrophil functionality.

Previous studies show that inhibition of PI3K or genetic deletion of Akt1 enhances LMMHA-induced apoptosis of neutrophils. Phosphorylation of p38 and ERK1/2 was significantly decreased in Akt1^−/−^ mice neutrophils treated with LMMHA [12]. Administration of 17β-estradiol after trauma-hemorrhage prevents phosphorylation of Kupffer cell p38 MAPK and normalizes Kupffer cell production of IL-6, TNF-α, MIP-1α, and CXCL-2. Further studies are needed to investigate whether 17β-estradiol prevents the increase in neutrophil infiltration and cytokine production via PI3K, Akt, and p38MARK after LMMHA administration. 

## 4. Materials and Methods 

### 4.1. Mice and Model Description

C57BL/6 mice aged 6–8 weeks were obtained from the Zhejiang Academy of Medical Sciences. All experiments were approved by the laboratory animals ethical committee of the Zhejiang University of Technology and followed the NIH guide for laboratory animals (NIH Publication No. 85–23, revised 1996) for the care and use of animals. Mice were maintained under specific pathogen-free conditions and given access to food and water. To obtain mice at known stages of the estrous cycle, vaginal smears were examined daily. Mice have 4 or 5-day estrous cycles, and only mice with at least two consecutive 4-day cycles were used. LMMHA (200 kDa, 65 mg/kg) was administered to the trachea with a microsprayer (Penn-Century, Wyndmoor, PA, USA) [16]. 

### 4.2. Ovariectomized (OVX)

The lower abdomen of ether-anesthetized mice was sliced, and ovaries were removed. The abdominal tissue was sutured, and an intramuscular injection of Pentantibiotic (570 mg/kg) was made. Vaginal smear cellular morphology was analyzed to determine the effectiveness of OVX after 7 days. After a recovery period of at least two weeks, OVX mice received subcutaneous injection of either vehicle (sesame oil, 2 mL/kg BW) or E2 (17β-estradiol, twice a day, 3 μg/kg BW) for one week. The experimental protocol was approved by the Laboratory Animals Ethical Committee of Zhejiang University of Technology (11 May 2020) and was registered on the Zhejiang University of Technology Animal Protocols Register (20200512053, 11 May 2020).

### 4.3. Bronchoalveolar Lavage Fluid (BALF)

Mice were sacrificed 12 h after intratracheal administration of LMWHA (200 kDa, 65 mg/kg). A midline incision was used to expose the trachea, which was then cannulated with a sterile 22-gauge needle. A total of 0.5 mL of cold PBS was injected and retrieved through the midline incision four times for a total of 1.8 mL BALF collected per mouse. The samples were then centrifuged, and sample supernatants of were stored at −80 °C. A hemocytometer was used to count total cell numbers in BALF samples. BALF neutrophil counts were determined using cytospin preparations stained with the Diff-Quick staining kit (IMEB, San Marcos, CA, USA).

### 4.4. ELISA

IL-6, TNF-α, CXCL-1, and CXCL-2 levels in BALF were quantified with mouse ELISA assay kits (R&D Systems, Minneapolis, MN, USA) as per the manufacturer’s protocol. 17β-estradiol levels in plasma were measured using a high sensitivity ELISA kit (ADI-900-174, Enzo Life Sciences).

### 4.5. Myeloperoxidase Assay

After BALFs were obtained, whole-lung homogenates were measured using a mouse myeloperoxidase (MPO) ELISA kit (Cell Sciences, Canton, MA, USA) per manufacturer protocol.

### 4.6. Lung Injury Score

Lung inflammation was assessed as described previously [10]. Blinded quantal injury assessment was performed through evaluation of alveolar septae, alveolar hemorrhage, intra-alveolar fibrin, and intra-alveolar infiltrates. A 10% buffered formalin was used to fix lung tissues overnight. These sections then underwent H&E staining: dehydration, clearing, and embedding in paraffin. At minimum, five fields were examined per section.

### 4.7. Statistical Analysis

Data from at least three independent experiments are expressed as mean ± SEM. In comparisons of two groups, a Student’s paired two-tailed *t*-test was used.

## 5. Conclusions

Our results illustrate the role of ovarian hormones and exogenous estrogen in modulation of lung injury and neutrophil accumulation in LMMHA-injured lungs. In addition, IL-6, CXCL-1, TNF-α, and CXCL-2 have been shown to enhance the inflammatory response in OVX and male mice. Finally, they show that 17β-estradiol can be considered as a therapy for reducing lung inflammation.

## Data Availability

The data that support the findings of this study are available from the corresponding author upon reasonable request.

## Abbreviations

LMMHA	Low molecular mass hyaluronan
IL-6	Interleukin-6
TNF-α	Tumor necrosis factor α
CXCL-1	Chemokine (C-X-C motif) ligand 1
CXCL-2	Chemokine (C-X-C motif) ligand 2
OVX	Ovariectomized
ALI	Acute lung injury
HA	Hyaluronan
BALF	Bronchoalveolar Lavage Fluid
MPO	Myeloperoxidase
E2	17β-estradiol
ARDS	Acute Respiratory Distress Syndrome
